# Venous thromboembolism and cancer risk

**DOI:** 10.1007/s11239-016-1411-y

**Published:** 2016-08-13

**Authors:** Per Sandén, Peter J. Svensson, Anders Själander

**Affiliations:** 1 Department of Public Health and Clinical Medicine, Umeå University, Sundsvall, Sweden; 2 Department for Coagulation Disorders, University of Lund, Malmö, Sweden; 3 Department of Clinical Sciences, Karolinska Institutet, Danderyd Hospital, Stockholm, Sweden

**Keywords:** Venous thromboembolism, Cancer, Warfarin, VTE

## Abstract

Cancer increases the risk of venous thromboembolism (VTE) and about 20 % of all VTE are associated with cancer. VTE can also be used as a marker for occult cancer. The objective was to examine the correlation between VTE and cancer regarding predictors for a subsequent cancer diagnosis. Patients treated for VTE between January 1st 2006 and December 31th 2011 were extracted from the Swedish national quality register AuriculA and crossmatched with the Swedish National Patient Register. In total 7854 patients corresponding to 14284 treatments years were examined. Primary VTE was found in 6451 patients, with 3936 first and 2515 recurrent VTE. There were 1403 patients with secondary VTE. After a first or recurrent primary VTE the incidence of cancer diagnose was high being 9.4–10.0 % the first year compared to 2.7–2.5 % during the second year. Cancer in the digestive organs was the most common type of cancer among those with first primary VTE with 19.2 % of diagnoses. In multivariable analysis age was found to increase the risk of cancer diagnosis after both first and recurrent primary VTE HR 1.02 (CI 1.02–1.03) and HR 1.02 (CI 1.01–1.03). For a first primary VTE anemia HR 2.13 (CI 1.48–3.08) and male sex HR 1.38 (CI 1.09–1.76) increased the risk while hypertension HR 0.74 (0.57–0.96), dementia HR 0.30 (CI 0.10–0.95) and history of major bleeding HR 0.52 (CI 0.28–0.97) reduced the risk of a subsequent cancer diagnosis. There is a substantial proportion of patients being diagnosed with cancer the first year after a primary VTE, anaemia and male sex confers an increased risk.

## Background

Cancer is known to increase the risk of venous thromboembolism (VTE) and about 20 % of all VTE are associated with cancer [1, 2]. Patients with cancer have a four to seven times increased risk of developing VTE and chemotherapy increases the risk further [3]. Tumours can express different procoagulant molecules and modify the expression of tissue factor which is one explanation to the increased risk of developing VTE among cancer patients [4]. Some tumours compress vessels altering blood flow or create damages to the vessel wall through intravascular growth increasing the risk for thrombosis.

VTE is also a marker for occult cancer [5], up to 10 % of patients with a first VTE have a subsequent cancer diagnosis within the first year [6, 7]. The risk of VTE differ between different cancer types [8], where cancer in the pancreas, kidney, stomach, ovary, brain and lymphomas have shown the strongest correlation to venous thrombosis. After a first VTE recurrence is relatively common and a concurrent cancer increases that risk [9]. Recurrent VTE has also previously been shown to increase the risk for a subsequent cancer diagnosis [10].

The aim of this study was to examine the correlation between VTE and cancer regarding predictors for a subsequent cancer diagnosis.

## Materials and methods

This is a retrospective study using secondary databases.

### Registries

AuriculA [11] was founded in 2006 and is a Swedish national quality register for atrial fibrillation (AF) as well as all patients on oral anticoagulation regardless of treatment indication. The register now includes over 125.000 patients and more than 7.000.000 INR values. Of all patients on oral anticoagulation in AuriculA, 19 % are treated due to a VTE. Approximately 50 % of all patients on warfarin in Sweden are included in AuriculA, mainly in whole regions with no apparent selection bias. Everything done with the patients in the anticoagulation centres in everyday clinical practice is recorded and transferred to the quality register automatically once every 24 h, provided that the patient has not declined to participate. AuriculA also provides a clinical decision tool, aiding in the dosage of warfarin using a dosing algorithm.

The Swedish National Patient Register [12] contains information about hospital admissions as well as visits in outpatient clinics in Sweden for all patients with a Swedish personal identity number. The register was launched in 1964, but complete coverage began in 1987. Currently, more than 99 % of all somatic and psychiatric hospital discharges are registered in the NPR. Information in this register includes dates for admission and discharge, ICD10 codes for primary and secondary diagnoses as well as surgical procedures, age and sex category.

The national quality register AuriculA was matched with the NPR at the Swedish national board of health and welfare using personal identity numbers and a common database was created which was anonymised. From this database patients treated with warfarin due to VTE between January 1´st 2006 and December 31´th 2011 with available information about background characteristics in the NPR were extracted.

### Patient population

In total 8831 patients with a first or recurrent VTE were retrieved. 8112 remained in the study after exclusion of those with a prior cancer diagnosis (n = 719). We analysed secondary VTE defined as patients with diagnosis of fracture or surgery within 6 months prior to warfarin initiation (n = 1403) separately. Patients with primary VTE with diagnosis between 90 days and 1 week before warfarin treatment were excluded from analysis (n = 258).

### Statistical analysis plan

A first VTE was defined as a first time diagnosis of a VTE within 1 week before start of warfarin treatment. Recurrent venous thrombosis was defined as presence of a previous VTE diagnosis 90 days or more before start of warfarin treatment due to the recurrent VTE. During the study period, almost all patients with VTE were treated with warfarin in Sweden, new oral anticoagulants were not available for this indication by then.

ICD10 codes were used for characterising background factors and new cancer diagnoses (Appendix).

Background characteristics were presented descriptively and differences between subgroups were tested with t-tests and X^2^-tests. Proportion diagnosed with cancer was described as diagnoses per 100 observation years expressed as percent.

Correlation between background factors and cancer diagnosis was analysed using multivariate cox regression analysis. Covariates were selected based on biological plausibility using ICD 10 codes defined in Appendix with the exemption of covariates with low a probability of registration in NPR.

Data was analysed using SPSS Statistics (Version 22; SPSS Inc., IBM Corporation, NY, USA), Confidence intervals (CI) are 95 % p < 0.05.

## Results

In total 3936 patients with a first, 2515 with a recurrent primary venous thrombosis and 1403 patients with a VTE secondary to surgery or fracture were examined, and followed for 14,284 patient years. Those with a recurrent thrombosis were older, 68,6 compared to 66,3 years (p = <0.001), and had a higher proportion of previous diseases (Table 1).

**Table 1:** Background characteristics for patients at initiation of warfarin treatment presented as n (%)

	First VTE	Recurrent VTE	Secondary VTE
	N = 3936	N = 2515	N = 1403
Start age (SD)	66.3 (15.8)	68.5 (15.2)**	66.4 (15.9)
Sex (female)	1879 (47.7)	1198 (47.6)	803 (57.2)
Hypertension	1297 (33.0)	1056(42.0)**	663 (47.3)
Cardiac failure	261 (6.6)	332(13.2)**	165 (11.8)
Myocardial infarction	272 (6.9)	288 (11.5)**	137 (9.8)
Stroke/TIA	405 (10.3)	330 (13.1)**	152 (10.8)
Diabetes	446 (11.3)	318 (12.6)	193 (13.8)
Thyroid disorder	203 (5.2)	166 (6.6)*	138 (9.8)
Chronic pulmonary disease	286 (7.3)	362 (14.4)**	164 (11.7)
Dementia	100 (2.5)	67 (2.7)	28 (2.0)
Anaemia	263 (6.6)	258 (10.3)**	180 (12.8)
History of major bleeding	203 (5.2)	217 (8.6)**	150 (10.7)
Renal failure	120 (3.1)	127 (5.1)**	92 (6.6)
Liver failure	58 (1.5)	30 (1.2)	28 (2.0)

Difference between first and recurrent VTE

* Significance with p < 0.05

** Significance with p < 0.001

The incidence of cancer diagnosis during the first year after initiation of warfarin was 9.4 % per year for those with a first VTE and 10.0 % for those with a recurrent VTE (Table 2). During the second year the incidence of a new cancer diagnose was 2.7 and 2.5 % for those with a first or recurrent VTE, respectively.

**Table 2:** Cancer diagnoses displayed as proportion (%) per observed time with 95 % CI, subdivided in primary VTE (first or recurrent) and VTE secondary to fracture or surgery

	Proportion diagnosed with cancer		
	Year 1	Year 2	Year 3
Primary VTE combined	9.6 (8.9–10.5)	2.7 (2.2–3.2)	2.0 (1.5–2.6)
First	9.4 (8.4–10.5)	2.7 (2.1–3.4)	1.9 (1.2–2.6)
Recurrent	10.0 (8.7–11.3)	2.5 (1.8–3.3)	2.2 (1.3–3.2)
Secondary VTE	2.5 (1.6–3.5)	0.4 (0–0.8)	1.1 (0.1–2.1)

After a secondary VTE, 2.5 % per year and 0.8 % per year had a new cancer diagnosis during the first or second year.

Almost all patients in the study, 96 %, were treated for venous thrombosis in the leg or lung.

The most common cancer type for those with a first VTE was cancer in the digestive organs (19.2 %) and cancer in the male genitals for those with recurrent VTE (23.8 %) (Table 3). Among men cancer in the male genitals was the most common type (35.8 %) of which 96 % were prostate cancer and among women it was cancer in the digestive organs (19.4 %).

**Table 3:** Type of cancer diagnosed during the first year after a primary VTE (first or recurrent). Results given as numbers and proportion (%) of total

Localisation	First VTE	Recurrent VTE
	N = 297	N = 202
Unspecified	64 (21.6)	40 (19.8)
Male genital organs	53 (17.9)	48 (23.8)
Digestive organs	57 (19.2)	32 (15.8)
Lymphoid hematopoietic and related tissue	46 (15.5)	38 (18.8)
Urinary tract	37 (12.5)	22 (10.9)
Respiratory and intrathoracic organs	30 (10.1)	12 (5.9)
Breast	20 (6.7)	17 (8.4)
Female genital organs	21 (7.1)	10 (5.0)
Eye, brain and other parts of central nervous system	13 (4.4)	5 (2.5)
Skin	6 (2.0)	10 (5.0)
Bone and articual cartilage	2 (0.7)	2 (1.0)
Mesotelial and soft tissue	3 (1.0)	2 (1.0)
Lip, oral cavity and pharynx	1 (0.3)	2 (1.0)
Thyroid and other endocrine glands	0	1 (0.5)

Correlation between a new cancer diagnosis the first year after a VTE and background factors was analyzed using multivariate Cox regression (Table 4). Factors that increased the risk of a cancer diagnosis during the first year for those with a first VTE were age HR 1.03 (1.02–1.03), anemia HR 2.13 (1.48–3.08) and male sex HR 1.38 (1.09–1.76). Hypertension HR 0.74 (0.57–0.96), dementia HR 0.30 (0.10–0.95) and history of major bleeding HR 0.52 (0.28–0.97) reduced the risk of a subsequent cancer diagnosis. For those with recurrent VTE only age HR 1.02 (1.01–1.03) significantly increased the risk of a new cancer diagnosis. For patients over 45 years old with a first VTE, age HR 1.02 (1.01–1.03), anemia HR 2.18 (1.50–3.16) and male sex HR 1.37 (1.07–1.75) increased the risk of a cancer diagnosis. Hypertension HR 0.73 (0.56–0.95), dementia HR 0.32 (0.10–1.00) and history of major bleeding HR 0.53 (0.29–0.99) reduced the risk of a subsequent cancer diagnosis.

**Table 4:** Multiple cox regression of background characteristics and the occurrence of a new cancer diagnosis during the first year after a first or recurrent primary VTE

	First VTE			Recurrent VTE		
	HR	p	95 % CI	HR	p	95 % CI
Start age	1.03	<0.001	1.02–1.03	1.02	0.001	1.01–1.03
Sex (male)	1.38	0.008	1.09–1.76	1.24	0.14	0.93–1.66
Hypertension	0.74	0.02	0.57–0.96	1.17	0.31	0.87–1.57
Cardiac failure	1.06	0.77	0.69–1.64	1.05	0.83	0.69–1.58
Myocardial infarction	0.63	0.07	0.38–1.04	0.61	0.06	0.37–1.02
Stroke/TIA	0.77	0.22	0.51–1.16	0.82	0.38	0.54–1.27
Diabetes	1.10	0.62	0.76–1.58	0.70	0.13	0.44–1.12
Thyroid disorder	1.14	0.61	0.69–1.87	0.76	0.39	0.41–1.42
Chronic pulmonary disease	1.34	0.14	0.91–1.99	1.37	0.09	0.95–1.99
Dementia	0.30	0.04	0.10–0.95	0.99	0.99	0.44–2.26
Anaemia	2.13	<0.001	1.48–3.08	1.04	0.86	0.67–1.63
History of major bleeding	0.52	0.04	0.28–0.97	1.45	0.09	0.94–2.24
Renal failure	1.26	0.45	0.70–2.27	1.34	0.30	0.77–2.33
Liver failure	0.0	0.92	–	1.39	0.58	0.44–4.41

## Discussion

After a first primary VTE a there is a high rate of cancer diagnosis with 9.4 % per year during the first year and additionally 2.7 % during the second year. For recurrent primary VTE the rate diagnosed with cancer is similar with 10.0 % during the first year, this surpasses the 2.5 % per year after a VTE secondary to fracture or surgery.

These results are well in line with those previously published where Sørenssen et al. [10] found an increased standardized incidence ratio of 2.2–3.2 for a cancer diagnosis the first year after a first or recurrent primary VTE, Prandoni et al. a frequency of 7.6–17.1 % [13] and Sun et al. [14] showing a clearly higher risk of cancer diagnosis after unprovoked VTE compared to a provoked VTE.

Except for rising age, which is a predictor for cancer diagnosis both after a first and recurrent VTE, we found anaemia to be a risk factor for a new cancer diagnosis after a first primary VTE in accordance to results by Trujillo-Santo et al. [15]. Anaemia has also been shown to increase the risk of VTE after cancer diagnosis [16] further supporting this connection. However, anaemia was not identified to be a risk factor for those with recurrent primary thrombosis, which to our knowledge has not been studied before. Those with a previous VTE have had treatment with anticoagulants where potential bleeding sources like tumours in the gastrointestinal tract might already have been discovered, rendering a new cancer diagnosis emerging after a recurrent VTE less likely. The same was true for patients with a history of major bleeding, where the risk of a new cancer diagnosis was reduced after a first but not after recurrent VTE, possibly due to the same mechanism as proposed above for anaemia. Major bleeding could cause immobilization and prolonged hospital stay, which increases the risk of VTE by itself, which also might explain the lower prevalence of new cancer diagnoses in this group.

Male sex increased the risk of obtaining a cancer diagnosis after a first primary VTE and a trend towards an increased risk for males with recurrent primary VTE. This has not previously been shown. Ihaddadene et al. [17] found no association between sex and occult malignancy, Sørenssen et al. [10] found no particular gender difference and Trujillo-Santos et al. [15] found an odds ratio of 1.3 for male sex but did not reach significance. The explanation for the increased risk of a cancer diagnosis associated with male sex found here is not fully understood. One reason could be that there are other causes of venous thrombosis in women like pregnancy or hormonal treatment for which we lack information in our material. However, limiting our analysis to patients above 45 years of age where hormonally induced VTE in women is unlikely, male sex is still a strong risk factor for a subsequent cancer diagnose. One recent study [18] found an increased risk for first time VTE among men compared to females if female reproductive factors are taken into account. Here they discussed differences in genetic factors as one possible explanation. The increased risk of new cancer diagnosis among males identified in our study could be an alternative explanation for part of the higher risk of VTE associated with male sex.

Hypertension reduced the risk of a cancer diagnosis after a first VTE and there was a trend towards a reduced risk after a myocardial infarction in both first and recurrent VTE. Interestingly, Lind et al. [19] have published an increased prevalence of myocardial infarction after VTE, showing a link between cardiovascular disease and VTE risk. With cardiovascular disease as an alternate cause of VTE concurrent cancer might less often be the cause of VTE among these patients.

Dementia was shown to reduce the risk of a cancer diagnosis for a first but not for recurrent VTE. This may be due to a reluctance of physicians to search for malignancies in patients with severe dementia.

The most common type of cancer diagnosed after a recurrent VTE was in the male genitals with 23.8 %. Of these 96 % were prostate cancers. Previous studies have shown a stronger correlation between prostate cancer and venous thrombosis compared to breast cancer [9], which could contribute to the risk increase for males.

With the high rate of cancer among VTE patients limited screening for occult cancer have been advised, including medical history, physical exam, basic bloodwork and chest radiograph. More extensive screening programs including CT scans or PET scans (fludeoxyglucose positron emission tomography) have not been found to be beneficial in identifying more occult cancers or reducing mortality [20–22] and is not advised as routine for all VTE patients.

### Limitations

Using registers make the results dependant on the validity of data in the registers. The validity of the NPR has previously been shown to be good. In somatic care the register lacks information in only 0.5–0.9 % of hospital admissions [23]. However, information about diagnoses from the primary health care is not included in the NPR, and therefore some concomitant diseases may have been underestimated. Cancer however is almost exclusively diagnosed and at least initially managed in specialised healthcare.

The inclusion of gender specific cancer complicates the analysis and may bias the results. However being a large proportion of all cancer the exclusion of gender specific cancer may also have created bias.

We also lack information about some factors that may influence both the cancer risk and risk for venous thrombosis like smoking, obesity and medical treatment. These could all be factors confounding the results. On the other hand, we here present a large real-life cohort of 7854 VTE patients corresponding to 14,284 observation years.

## Conclusion

There is a substantial proportion of patients being diagnosed with cancer the first year after a primary VTE, anaemia and male sex confers an increased risk. Screening for cancer after a VTE has previously not been advised, but physicians caring for patients with VTE should be aware of symptoms and findings indicating occult cancer, especially among men and elderly without cardiovascular morbidity.

## Appendix

**Table:**

ICD-10 Codes	
Background characteristics	
Hypertension	I10–15
Cardiac failure	I50, I110, I130, I132
Myocardial infarction	I21–22, I252
Stroke/TIA	I63–64, G450, G451, G452, G453, G458, G459, I69
Diabetes	E10–14
Thyroid disorder	E00–E07
Chronic pulmonary disease	J40–70
Dementia	F00–03
Anemia	D50, D52, D53, D55, D59, D60, D61–D64, D510, D513, D518, D519, D560–D562, D568–D572, D588, D589.
Major bleeding	I60, I61, I62, R04, K250, K252, K254, K256, K260, K262, K264, K266, K270, K272, K274, K276, K280, K282, K284, K286, K922, K290, I850, I983, K625, D629, R589
Renal failure	I120, I131–132, N17–19, DR016, DR024, KAS00, KAS10, KAS20
Liver failure	K70–77, JJC, JJB
Cancer	C00–C97
